# Understanding the Genomic Structure of Copy‐Number Variation of the Low‐Affinity Fcγ Receptor Region Allows Confirmation of the Association of *FCGR3B* Deletion with Rheumatoid Arthritis

**DOI:** 10.1002/humu.23159

**Published:** 2017-02-15

**Authors:** Raheleh Rahbari, Luciana W Zuccherato, German Tischler, Belinda Chihota, Hasret Ozturk, Sara Saleem, Eduardo Tarazona‐Santos, Lee R Machado, Edward J Hollox

**Affiliations:** ^1^ Department of GeneticsUniversity of LeicesterLeicesterUnited Kingdom; ^2^ Wellcome Trust Sanger InstituteHinxtonUnited Kingdom; ^3^ Departmento de Biologia GeralInstituto de Ciências BiológicasUniversidade Federal de Minas GeraisBelo HorizonteBrazil; ^4^ School of HealthUniversity of NorthamptonNorthamptonUnited Kingdom

**Keywords:** Fcγ receptors, FCGR3B, CNV, copy‐number variation, deletion, rheumatoid arthritis

## Abstract

Fcγ receptors are a family of cell–surface receptors that are expressed by a host of different innate and adaptive immune cells, and mediate inflammatory responses by binding the Fc portion of immunoglobulin G. In humans, five low‐affinity receptors are encoded by the genes *FCGR2A*, *FCGR2B*, *FCGR2C*, *FCGR3A*, and *FCGR3B*, which are located in an 82.5‐kb segmental tandem duplication on chromosome 1q23.3, which shows extensive copy‐number variation (CNV). Deletions of *FCGR3B* have been suggested to increase the risk of inflammatory diseases such as systemic lupus erythematosus and rheumatoid arthritis (RA). In this study, we identify the deletion breakpoints of *FCGR3B* deletion alleles in the UK population and endogamous native American population, and show that some but not all alleles are likely to be identical‐by‐descent. We also localize a duplication breakpoint, confirming that the mechanism of CNV generation is nonallelic homologous recombination, and identify several alleles with gene conversion events using fosmid sequencing data. We use information on the structure of the deletion alleles to distinguish *FCGR3B* deletions from *FCGR3A* deletions in whole‐genome array comparative genomic hybridization (aCGH) data. Reanalysis of published aCGH data using this approach supports association of *FCGR3B* deletion with increased risk of RA in a large cohort of 1,982 cases and 3,271 controls (odds ratio 1.61, *P* = 2.9×10^−3^).

## Introduction

Fcγ receptors are a family of cell–surface receptors that are expressed by a host of different innate and adaptive immune cells, and mediate inflammatory responses by binding the Fc portion of immunoglobulin G (IgG) [Nimmerjahn and Ravetch, 2008]. They can be divided into low‐ and high‐affinity receptors, based on their affinity for IgG. Low‐affinity receptors are unable to bind monomeric IgG and instead bind to polymeric IgG in the form of antigen–antibody immune complexes. IgG binding can either activate or inhibit downstream cellular responses depending on the particular ITAM or ITIM containing Fcγ receptor that is engaged. Dysregulation of Fcγ receptors is important in a number of different inflammatory diseases, including rheumatoid arthritis (RA), systemic lupus erythematosus (SLE), and Kawasaki disease [Niederer et al., 2010a; McKinney and Merriman, 2012; Hargreaves et al., 2015b]. Furthermore, not only are Fcγ receptors critical for disease etiology but also for successful immunotherapy of patients with hematological and solid cancers by mediating the effector functions of therapeutic monoclonal antibodies [Dahal et al., 2015].

**Figure 1:**
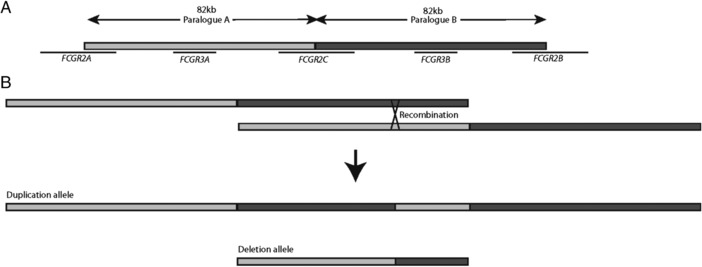
Structure of low‐affinity Fc γ receptor region. **A**: Two 82 kb paralogs repeats with 98.5% identity at chromosome 1p23.3 carry the low‐affinity Fc γ receptor genes *FCGR2A*, *FCGR3A*, *FCGR2C*, *FCGR3B*, and *FCGR2B*. **B**: Nonallelic homologous recombination between paralogs can generate deletion and duplication alleles where the duplicated allele contains a chimeric copy.

In humans, there are five low‐affinity receptors FcγRIIa, FcγRIIb, FcγRIIc, FcγRIIIa, and FcγRIIIb encoded by the genes *FCGR2A* (MIM# 146790), *FCGR2B* (MIM# 604590), *FCGR2C* (MIM# 612169), *FCGR3A* (MIM# 146740), and *FCGR3B* (MIM# 610665). All five genes are encoded within or surrounding an 82.5‐kb tandemly arranged segmental duplication on chromosome 1q23.3 (Fig. 1). There is substantial single‐nucleotide variation within and between the duplicated paralogs, and extensive copy‐number variation involving *FCGR3A*, *FCGR3B*, and *FCGR2C* [Aitman et al., 2006; Hollox et al., 2009; Machado et al., 2012; Mueller et al., 2012]. The extent of variation has made dissecting the genomic structure of this region particularly challenging. It is now generally accepted that copy‐number variation is a result of more or fewer copies of the full 82.5 kb segmental duplication, resulting in the deletion or duplication of *FCGR3A* and *FCGR2C* together, or *FCGR3B* and *FCGR2C* together, but not *FCGR2A* or *FCGR2B*, which fall outside the CNV region [Breunis et al., 2009; Hollox et al., 2009; Mueller et al., 2012]. The mechanism generating *FCGR* deletions and duplications has been shown to be mediated by nonallelic homologous recombination (NAHR) between the two segmental duplications with the location of the breakpoint determining whether *FCGR3A* or *FCGR3B* genes are deleted, and whether fusion *FCGR2A/C* genes are generated [Machado et al., 2012; Nagelkerke et al., 2015]. The breakpoints of several deletion alleles have been determined and, although resolution to the exact nucleotide is not possible, breakpoints cluster at two points: breakpoint A [Machado et al., 2012], generating CNR3 [Nagelkerke et al., 2015]; and breakpoint B, generating CNR1. Rarer breakpoints, termed CNR2 and CNR4, have also been observed, and generate *FCGR2B* pseudogenes and *FCGR2C/A* fusion genes [Machado et al., 2012; Nagelkerke et al., 2015]. The accuracy of some of the breakpoint locations is still uncertain; while a precise localization of each breakpoint is usually impossible, analysis of fosmid clones from multiple individuals has suggested a complete absence of paralog sequence variants (PSVs, i.e., sequence variants that can always distinguish one paralog from another) in the distal 24.5 kb of each repeat that effectively prohibits higher‐resolution breakpoint mapping in that region [Mueller et al., 2012].

In this context, genetic association studies at this locus that involve genotyping copy number, sequence variants, or both, can often be unreliable due to a combination of technical variation due to noisy assays measuring copy‐number variation and biological variation because of the underlying complexity of the locus [Haridan et al., 2015; Hargreaves et al., 2015a]. Improved assays based on the paralog ratio test (PRT) [Armour et al., 2007] and multiplex ligation probe amplification (MLPA) [Schouten et al., 2002] have allowed some studies to suggest an association of the *FCGR3B* deletion with SLE [Morris et al., 2010; Niederer et al., 2010b] and RA [Robinson et al., 2012]. Importantly, these assays allowed the distinction of *FCGR3B* copy number from *FCGR3A* copy number, although it should be noted that MLPA does not distinguish the nucleotide change that defines *FCGR3A* from *FCGR3B*. In contrast, the largest study used array comparative genomic hybridization (aCGH) to call copy number at this locus in the Wellcome Trust Case Control Consortium (WTCCC) and failed to find an association [Craddock et al., 2010]. Crucially, an analysis of the aCGH data did not distinguish between deletions of *FCGR3A* and *FCGR3B*, which would contribute to a lack of power to observe such an association.

In this study, we develop our understanding of the variation within this region by reanalyzing the fosmid sequences previously generated in the light of published findings [Machado et al., 2012; Nagelkerke et al., 2015]. We investigate the role of gene conversion in this region and identify new gene conversion alleles. We also show how awareness of the structure of deletions in this region can distinguish *FCGR3A* deletions from *FCGR3B* deletions in aCGH data. This allows us to reanalyze aCGH data from RA cases and healthy controls, and test the association of the *FCGR3B* deletion with RA.

## Methods

### Samples and Data

Four‐hundred eighty British samples were obtained from the Human Random Control series (Public Health England, Porton Wiltshire, UK). Native American samples were obtained from the Ashaninka population from five villages along the Junin River in Central Peru or from the Matsiguenga population living in the community of Shimaa, Peru. The Ashaninkas and Matsiguengas live in the Amazon Yunga tropical forest, and speak languages belonging to the Arawak family. DNA collection was collected under informed consent and approved by local ethics institutional review boards. WTCCC data were provided courtesy of the WTCCC Access Committee and Dr. Matthew Hurles (Wellcome Trust Sanger Institute). Full details of the cohorts have been published previously [Craddock et al., 2010]. 454 technology‐based sequencing reads were downloaded from the European Nucleotide Archive (accession number ERA168468) in the BAM format. Details of the samples analyzed are given in Table 1.

**Table 1:** Samples Previously Sequenced Across the Fcγ Receptor Region

Fosmid library	Population	Sample ID	Copy number	A copy number	B copy number
ABC7	YRI	NA18517	3	1	2
ABC8	YRI	NA18507	4	2	2
ABC9	JPT	NA18956	5	2	3
ABC10	YRI	NA19240	4	2	2
ABC11	CHB	NA18555	5	2	3
ABC12	CEU	NA12878	4	2	2
ABC13	YRI	NA19129	4	2	2
ABC14	CEU	NA12156	4	2	2

### 454 Sequence Assembly and Alignment

For each fosmid, the reads were mapped to the cloning vector sequence (cloning vector pEPIFOS‐5) using version 0.7.6 of the SMALT aligner. Reads mapping to the vector with an alignment score of less than 30 were retained, and the rest were discarded as it was assumed they were contaminated with vector sequence. Filtered reads were assembled using version 2.9 of the GS De Novo Assembler (newbler). The assembled contigs labeled as large by the assembler were retained. Filtered reads were mapped to these contigs, and the consensus algorithm of GAP5 version 2.0.0b10 [Bonfield and Whitwham, 2010] was used to recompute the contig consensus sequences. Paralogs A (chr1:159815745‐159831746) and B (chr1:159897573‐159913518) were extracted from the human reference genome hg18. The assembled contigs were mapped to paralogs A and B using the MEM algorithm of the Burrows‐Wheeler aligner [Li, 2013]. Alignments spanning less than 400 bases on the reference were discarded. For each sample and paralog, a maximal nonoverlapping set of alignments was kept and the rest of the alignments were discarded. The sequence identity (fraction of matching aligned bases) between the sample and reference paralogs was computed using the aligned contigs, and each contig was assigned to the paralog of the highest sequence identity. For each fosmid, the contigs chosen for the paralog assigned via identity were extracted in reference order and concatenated to obtain a single sequence representing the fosmid. Sequence from fosmids including sequence from the distal 16 kb region of either reference B or reference A, excluding alignment gaps, was used to compute a multiple sequence alignment by Clustal Omega [Sievers et al., 2011]. The multiple sequence alignment was used to construct a median network by SplitsTree version 4.14.2 [Huson and Bryant, 2006].

### FCGR 3 Copy Number Typing and Breakpoint Sequencing

Following *FCGR3* typing using a PRT/REDVR approach, as described previously [Hollox et al., 2009], two homozygous deleted individuals were identified from 480 UK individuals and selected for further analysis. PCR products spanning the region, as previously described [Machado et al., 2012], were end‐sequenced to determine the approximate location of breakpoints. PCR products spanning the breakpoint region were comprehensively sequenced by Sanger sequencing.

### aCGH Analysis

Quantile‐normalized log ratio data from Agilent 2×105K aCGH experiments were used [Conrad et al., 2009; Craddock et al., 2010]. Including technical duplicates, a total of 21,858 aCGH experiments were analyzed using the software CNVtools 1.42.3 [Barnes et al., 2008] implemented in R v.3.1.1 [The R Development Team, 2013]. Briefly, the first principal component (PC) of the data is fitted to a Gaussian mixture model, with three components, corresponding to normal, deletion, and duplication. Cohort is included as a factor in the mixture model to minimize differential bias in calling between cohorts. The mixture model then allows calling of copy number combined with a posterior probability of that call. A total of 1,363 samples with a deletion were called, and a subset verified against samples also typed by the paralog ratio test.

PC analysis on a narrower set of aCGH probes (see *Results*) was conducted using CNV tools on RA and control individuals showing heterozygous deletion, and a Gaussian mixture model run with two components to attempt to stratify *FCGR3B* deletions and *FCGR3A* deletions on the basis of values on the first PC, and case/control status included as a factor in the mixture model to minimize differential bias in calling between cohorts. In all analyses, the values from each aCGH probe were weighted equally.

### Association Analysis

Odds ratios and significance of departure from the null hypothesis were calculated from two‐by‐two contingency tables using Fisher's exact test, implemented in R.

## Results

### De Novo Assembly of High‐Throughput Sequencing Reads Supports a Simple NAHR Model for CNV and Patches of Gene Conversion

**Figure 2:**
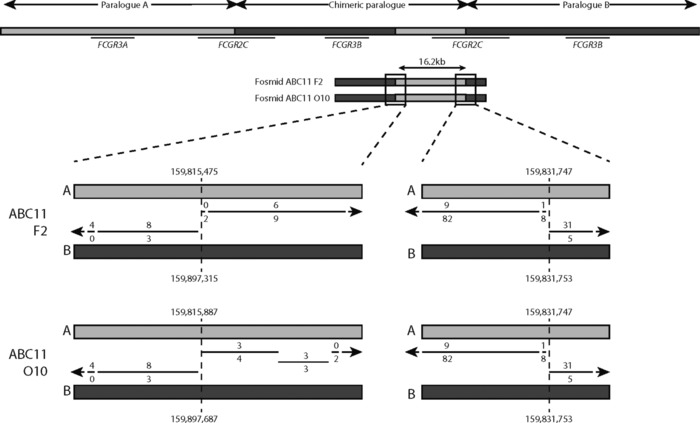
Duplication NAHR breakpoints in fosmid sequences. Duplications formed by NAHR will result in a chimeric paralog containing A and B sequences. Sample ABC11 (NA18555) is heterozygous for *FCGR3B* duplication. Two fosmids, both generated from one allele, show the NAHR breakpoint. The inferred location of the breakpoint (hg18) is shown, and apparently differs by 412 bp only because of the different sequence contigs constructed (see *Methods*). The lines show alignment of the fosmid contig sequences to A and B reference sequences, with the number of mismatches to A and B shown above and below the line, respectively.

Cloning diploid genomes into large‐ and medium‐size insert vectors, such as fosmids, provides an approach to characterize medium‐scale structural variation, such as the copy‐number variation involving the low‐affinity Fcγ receptor region. Previously, fosmid libraries were generated for eight individuals from the HapMap collection, and fosmids mapping to the Fcγ region fully sequenced using 454 sequencing technology [Kidd et al., 2008; Mueller et al., 2012]. After de novo assembly of contigs, the sequence reads were mapped directly to the reference genome. It had been suggested that further resolution of the region where most *FCGR3B* deletion breakpoints mapped was limited by the lack of sequence variants distinguishing A paralog sequences from B paralog sequences [Mueller et al., 2012]. It was therefore important to investigate this before pursuing further breakpoint mapping in this region. We used a novel approach to assign sequence contigs, which together comprised a fosmid sequence, to either A or B paralog sequences, and to investigate potential switches between A‐like and B‐like sequences within a fosmid. Mapping of the whole fosmid allowed assignment to a two copy, one copy, or three copy chromosome depending on the genotype of individual from whom the fosmid library was derived. Our fosmid mapping locations matched all those previously published [Mueller et al., 2012]. Because some of the fosmids overlapped with single copy sequence flanking the duplicated region, this mapping information provided a further check that our fosmid assignment to A (proximal) or B (distal) paralog was correct. We confirmed two clones to be from the duplicated allele of sample ABC11 (NA18555), and confirm that these are the reciprocal products expected from a model of NAHR, with a breakpoint similar to previous deletion breakpoints (Fig. 2).

**Figure 3:**
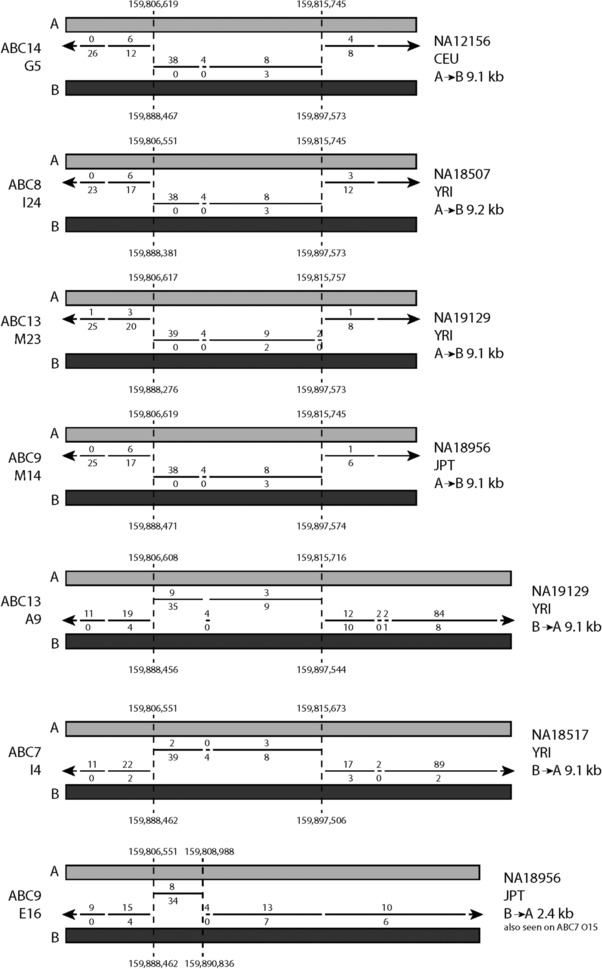
Gene‐conversion alleles identified in fosmid sequences. Representative fosmids of seven alleles carrying gene conversions are shown. Inferred breakpoints relative to hg18 paralog A and paralog B are shown. The lines show alignment of the fosmid sequences to A and B reference sequences, with the number of mismatches to A and B shown above and below the line, respectively. The first four alleles are identical, showing a 9.1‐kb B sequence on the A paralog. The following two show the reciprocal event, a 9.1‐kb A sequence on the B paralog. The final example shows a 2.4‐kb B sequence on an A paralog.

**Figure 4:**
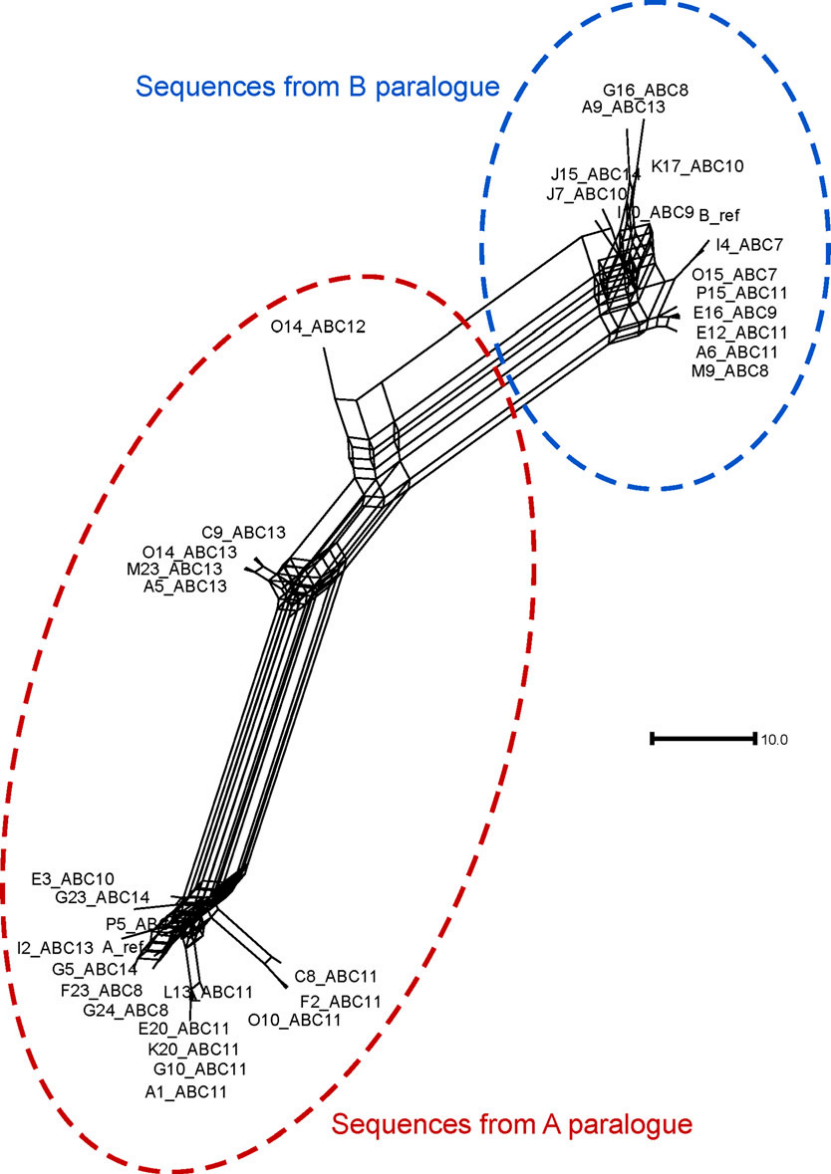
Median network of distal 16 kb fosmid sequences from A and B paralogs. Thirty‐three fosmid sequences corresponding to the distal 16 kb of both A and B paralogs were aligned, together with the hg18 human reference sequences for the A and B paralogs, and their relationships plotted as an unrooted median network. Sequences that are from fosmids mapping to the A paralog and B paralog are highlighted in the ellipses. Scale bar indicates 10 single‐nucleotide substitutions.

We observed evidence of gene‐conversion alleles by examining fosmid sequences. Three different alleles were initially found: an A to B gene conversion of about 9.1 kb (NC_000001.9:g.159806619_159815757con159888461_159897571), the reciprocal B to A gene conversion of 9.1 kb (NC_000001.9:g.159888461_159897571con159806619_159815757), and a smaller B to A gene conversion of 2.4 kb (NC_000001.9:g.159888397‐159890836con159806551_159808988). All three gene conversions occur between *FCGR3A/B* and *FCGR2B/C* in the region previously identified as a deletion breakpoint region [Machado et al., 2012; Mueller et al., 2012; Nagelkerke et al., 2015] (Fig. 3; Table 2). While these events would explain the proximal part of the 24.5 kb that was previously suggested to show no nonpolymorphic PSVs, we sought to investigate the remaining diversity of the distal ∼16kb. We therefore took the sequences corresponding to this region from the fosmids that we assembled, for both A and B paralogs, aligned them together, and constructed a median network from the alignment (Fig. 4). From this network, it is clear that sequences that derive from the A paralog are clearly distinguishable from the sequences that derive from the B paralog. Five A paralog fosmid sequences, from two alleles from samples ABC12 (NA12878) and ABC13 (NA19129), share more sequence with the B repeat sequences than other A paralog sequences. Inspection of our contig alignment calls suggests that O14_ABC12 has a fourth gene‐conversion allele that converts A sequences to B‐like sequence for about 7 kb, although the region cannot be well defined due to extensive sequence similarity in the region between the two paralogs (which we call an indistinct gene‐conversion allele; Table 2). The sequences from ABC13 fosmids that are distinct from the main A paralog cluster show a 6.4‐kb patch of sequence highly diverged from both A and B paralog reference sequences (which we call a highly diverged region; Table 2).

**Table 2:** Chromosomal Locations of Genomic Features Described in This Paper

Feature	Location (hg18)^a^
9.1 kb gene‐conversion alleles	NC_000001.9:g.159806619_159815757con159888461_159897571
	NC_000001.9:g.159888461_159897571con159806619_159815757
2.4 kb gene‐conversion allele	NC_000001.9:g.159888397_159890836con159806551_159808988
Indistinct gene‐conversion allele	Chr1:159824746–159831747
Highly diverged region	Chr1:159824223–159830623
Region used for multiple alignment and phylogenetic network	Chr1:159815745–159831746
UK deletion breakpoint regions	Chr1: 159801233–159801500
	Chr1: 159823772–159825582
	Chr1: 159805059–159805132
Native American deletion breakpoint regions	Chr1: 159775888–159775907
	Chr1: 159809793–159821996
	Chr1: 159811450–159823429

a Regions are given on the A paralog, but depending on the nature of the feature, the corresponding B paralog may also be affected. See text for details.

Although these gene‐conversion alleles may complicate assessment of breakpoint position in certain circumstances, the phylogenetic network (Fig. 4) shows that A paralog sequences are clearly distinguishable from B paralog sequences. This shows that A and B sequences can be distinguished by sequence differences alone, which does not support the observation of the 24.5‐kb region of haplotype swapping model previously proposed. The data support our sequence‐based approach for mapping NAHR breakpoints in the region.

### Analysis of Deletion Breakpoints in British and Shimaa Populations Show Population‐Specific Breakpoints

Previously, mapping deletion breakpoints in a globally diverse selection of alleles suggested that different deletion breakpoints were distinct yet clustered into two major hotspot regions [Machado et al., 2012]. Given that each one was distinct, this strongly suggested identity‐by‐state rather than identity by descent. However, it may be the case that within a single population, all deletion alleles are identical by descent because of the closer genetic ancestry between members of the same population. To answer this question, we screened three distinct populations for individuals homozygous for either *FCGR3A* deletion, *FCGR3B* deletion, or heterozygous for *FGCR3A* and *FCGR3B* deletions. We chose the British as one population, which is an outbred population at the edge of northwest Europe, for two reasons. First, we had not previously mapped a European deletion allele, yet most genetic association studies involving the *FCGR3B* deletion alleles are on European populations [McKinney and Merriman, 2012]. Second, many studies of disease association at this locus are on British cohorts, or cohorts with British ancestry [Bournazos et al., 2010; McKinney et al., 2010; Morris et al., 2010; Niederer et al., 2010b; Robinson et al., 2012]. Understanding the structure of the different deletion alleles will allow distinction of alleles that are likely to be identical by descent from those that are identical by state, and potentially allow refinement of disease associations.

**Figure 5:**
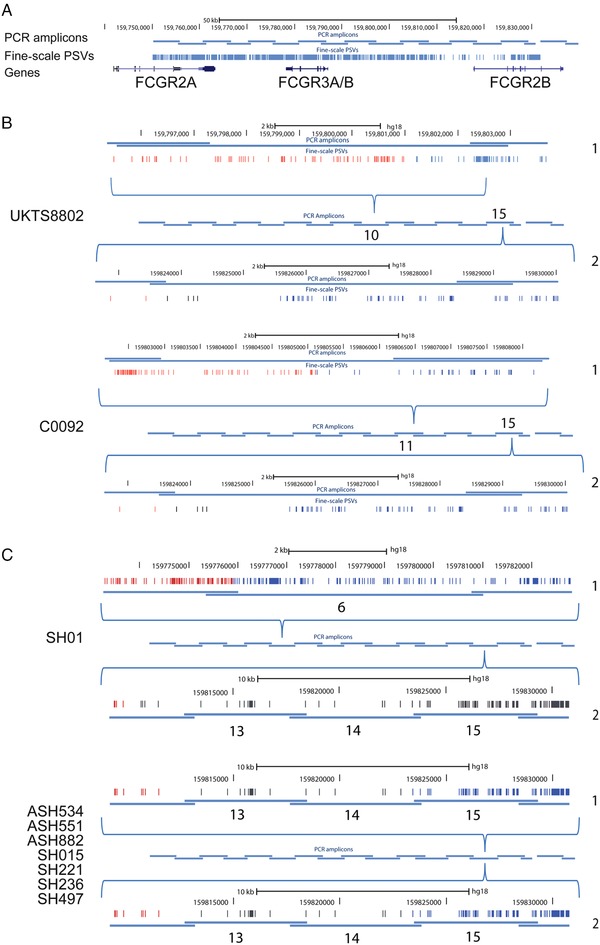
Fcγ receptor deletion breakpoint mapping. **A**: Schematic of PCR amplicons used to amplify the Fc γ receptor region in homozygous deletion individuals, as previously carried out [Machado et al., 2012]. **B**: Breakpoint mapping of two British individuals (UKTS8802 and C0092), homozygous for *FCGR3B* deletion. For each individual, both alleles (labeled as 1 and 2, on the right of each diagram) are shown. The overlapping PCR amplicons spanning the duplication are shown in the center, with the amplicon number showing the amplicon in which that particular breakpoint was found. Each amplicon containing the breakpoint is expanded (shown by the blue brackets above and below the PCR amplicons), showing the position of the PSVs as well as the hg18 chromosome 1 coordinates. A change in color from red (A‐like) to blue (B‐like) indicates the location of the breakpoint. If the breakpoint position is not clear, due to gene‐conversion events or possible recurrent NAHR events, the PSVs in the putative breakpoint region are colored black. **C**: Breakpoint mapping of eight native American individuals, seven homozygous for *FCGR3B* deletion, and one heterozygous for *FCGR3A* deletion and *FCGR3B* deletion. Individuals prefixed SH are Matsiguenga individuals, and those prefixed ASH are Ashaninka individuals.

Using PRT, we identified two UK samples that were homozygous for the *FCGR3B* deletion. Sequence analysis showed that both were heterozygous for the NAHR breakpoint, with one allele showing a breakpoint in region B, generating CNR1, and the other in a region distal to breakpoint B, generating CNR4 (Fig. 5; Table 2). The breakpoint generating CNR4 was particularly difficult to define due to the lack of informative variable sites. Using an eight‐SNP haplotype we have previously defined [Machado et al., 2012] (Supp. Tables S1 and S2) that is just distal to the CNV region, both UK samples proved to be heterozygous for two common haplotypes. Taken together, this strongly suggests that these *FCGR3B* deletion alleles are identical‐by‐state, but have been generated by at least three independent mutational events (Supp. Fig. S1).

We also chose the Shimaa and Ashaninka—two isolated native American groups resident in Peru that have an unusually high frequency of *FCGR3B* deletion alleles (27% in Shimaa, 18% in Ashaninka) [Zuccherato, 2012]. We selected seven individuals who were homozygous for *FCGR3B* deletions, and one individual who carried both a *FCGR3A* deletion and a *FCGR3B* deletion. Using a PCR amplification and Sanger sequencing approach, we located all the deletion breakpoints from the *FCGR3B* deletion homozygotes to a 10‐kb region immediately distal to the gene‐conversion region (Fig. 5). Because of the scarcity and ambiguity of PSVs within this region, particularly in native Americans, we could not refine this breakpoint further, although a breakpoint in this region has been previously observed in other populations [Machado et al., 2012]. All seven *FCGR3B* breakpoints were homozygous and identical for variant sites within this region strongly suggesting that the alleles in these populations were identical by descent. In the individual who carried a *FCGR3A* deletion and a *FCGR3B* deletion, the *FCGR3B* deletion breakpoint was mapped to the same region as the others, but a gene conversion event on one of the two homologous chromosomes obscured the breakpoint region. The *FCGR3A* deletion mapped to a region previously identified as breakpoint A [Machado et al., 2012], consistent with previous studies. Analysis of the eight‐SNP haplotype showed that all individuals were homozygous for the common haplotype in South Americans (Supp. Tables S2 and S3). Because of this, we cannot distinguish identity‐by‐state from identity‐by‐descent on eight‐SNP haplotype data alone.

### Reanalysis of aCGH Data Allows Discrimination of *FCGR3A* and *FCGR3B* Deletions

**Figure 6:**
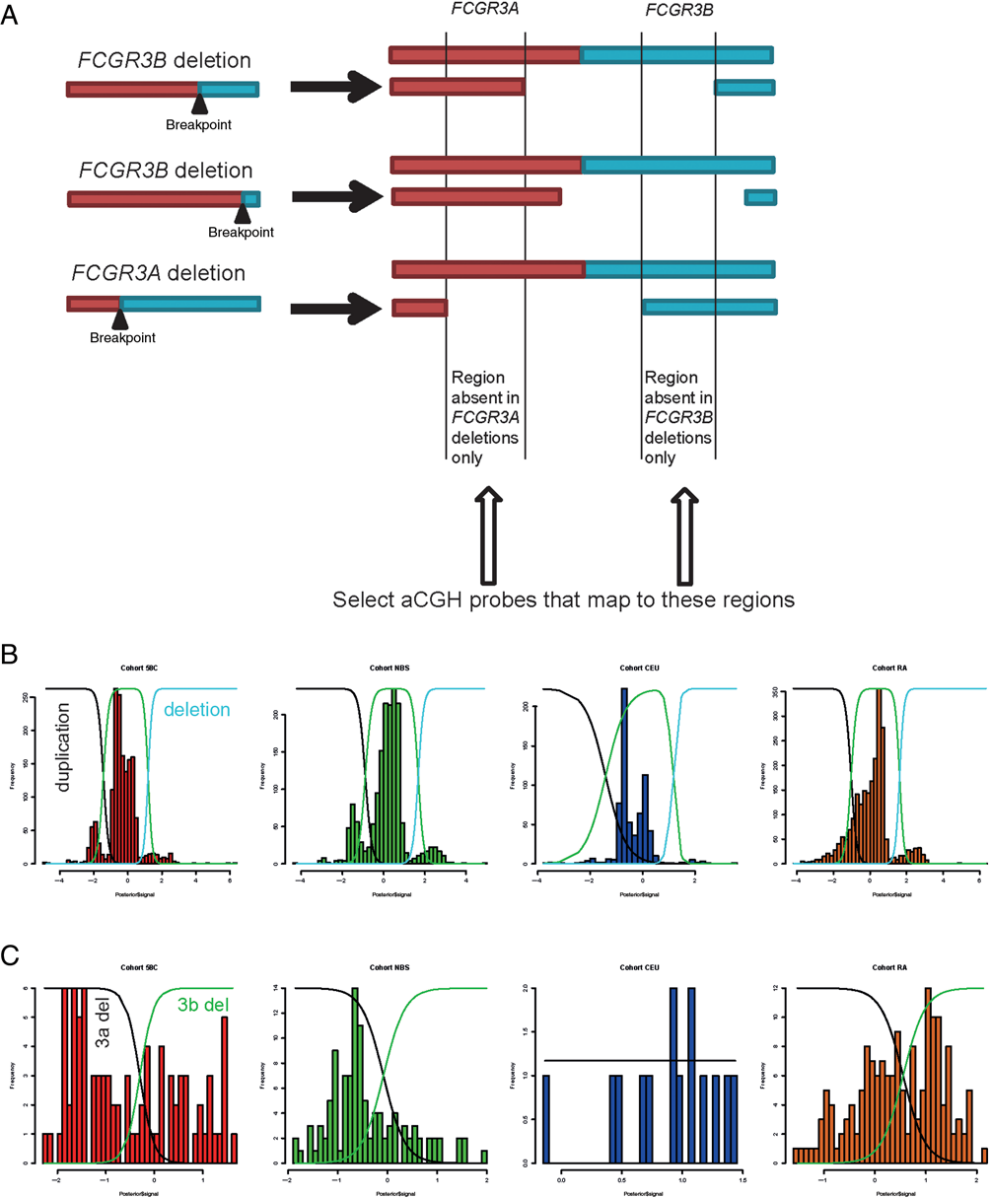
Analysis of Fcγ receptor region using array CGH data. **A**: The principle of selecting aCGH probes that are more likely to distinguish between *FCGR3A* and *FCGR3B* deletions. **B**: Clustering of aCGH data into deletion, duplication, and normal copy‐number clusters using Gaussian mixture modeling. Cohort labels: 58C, 1,958 cohort; NBS, National Blood Service cohort; CEU, CEPH Europeans from Utah; RA, rheumatoid arthritis cohort. **C**: Separation of deletion heterozygotes into deletions of *FCGR3B* and *FCGR3A* by fitting two Gaussian curves to the data.

Based on the data presented here and previously, it is a reasonable assumption that all *FCGR3B* deletion alleles share the same or very similar NAHR breakpoints at one or more NAHR hotspots at the distal end of the paralogs. Previous work, together with a single observation in this work, has shown that *FCGR3A* deletion alleles also share a breakpoint hotspot [Machado et al., 2012]. If we represent heterozygous individuals for these deletions (Fig. 6A), it can be seen that all heterozygous deletion genotypes will have a common region absent, but will also have regions absent uniquely in *FCGR3B* deletions and uniquely in *FCGR3A* deletions. We can also propose that the effectiveness of hybridization of particular aCGH probes that were designed to the A or B paralogs region will be influenced not only by total copy number of the duplicated region but also by sequence differences between *FCGR3A* copy and *FCGR3B* copy of the paralogs region, because of the sequence differences in those aCGH probes. Probes designed to hybridize to *FCGR3A* will also hybridize to *FCGR3B*, but more weakly, and vice versa.

We therefore initially called copy number of *FCGR3A* and *FCGR3B* in samples across the entire WTCCC dataset, and the CEU HapMap cohort included in the same aCGH experiment using 33 probes spanning both A and B paralogs. We then used a Gaussian mixture model to categorize samples into those carrying duplications, deletions, and those with the normal diploid copy number of two *FCGR3B* genes and two *FCGR3A* genes (Fig. 6B). It should be noted that the data quality, reflected in the extent to which different copy numbers clustered in the histogram, varied between different cohorts and, for this reason, only three components of the mixture model were used representing loss, normal or gain of copy number.

We then selected 20 aCGH probes that are a subset of the original 33 probes and that map only to the *FCGR3A* and *FCGR3B* deletion‐determining regions (Fig. 6A). We selected the individuals that showed deletion copy‐number values in the control and RA case cohorts (a total of 388 individuals comprising 170 RA cases, 204 controls, 14 CEU samples). We took these individuals and removed 13 individuals with extremely low copy number (PC1 value>3.8, seven from the cases, six from the controls) who will be deletion homozygotes and therefore could not be categorized into *FCGR3A* or *FCGR3B* deletions using our approach (Fig. 6B). We then applied PC analysis on the raw aCGH data from the remaining 375 individuals, expecting that because they have the same copy number, the probe intensity variation represented by the first PC will now be primarily due to sequence differences between *FCGR3B* deletions and *FCGR3A* deletions. On plotting the PC1 as a histogram, most samples cluster as a single group to the right of the plot with a few outliers to the left, which on larger cohorts begin to form a distinct peak. Using a Gaussian mixture model approach to split the data, we categorize the deletion samples into two groups that, based on 100% concordance with 15 known *FCGR3B* and *FCGR3A* deletions typed using PRT/REDVR, represent *FCGR3A* heterozygous deletions (Fig. 6C) and *FCGR3B* heterozygous deletions (Fig. 6C).

### RA Is Associated with *FCGR3B* Deletion

Using the approach above, we called heterozygous *FCGR3B* deletion individuals for the RA cohort and the control cohorts (Table 3; Supp. Table S4). Comparison of deletion heterozygote frequencies in cases and controls supported the association of RA with the *FCGR3B* deletion allele (OR 1.61, 95% CI 1.16–2.24, *P* = 2.9×10^−3^, Fisher's exact test).

**Table 3:** Association Analysis of *FCGR3B* Deletion and Rheumatoid Arthritis

Genotype	RA	Control	OR (95% confidence interval)	*P* value
*FCGR3B* deletion carriers	79	82	1.61 (1.16–2.24)	2.9×10^−3^
Noncarriers	1,903	3,189		

## Discussion

Our data show that for the two UK samples homozygous for the *FCGR3B* deletion (CO092 and UKTS8802), the breakpoints all map to the same regions as previously identified [Machado et al., 2013; Nagelkerke et al., 2015]. However, two of the UK deletion alleles appear to have the same breakpoints, suggesting that these deletion alleles are identical‐by‐descent. The other two UK deletion alleles show different deletion breakpoints, are therefore identical‐by‐state, and are likely to have been generated by recurrent mutation. This is supported by a mutation rate estimated at between 0.03% and 0.14% per generation at this locus [Machado et al., 2012]. In two endogamous populations from Peru, where a high frequency of *FCGR3B* deletion alleles has been reported [Zuccherato, 2012], we found that seven out of eight *FCGR3B* deletion breakpoints were identical, showing that these deletions in these populations are identical by descent. Therefore, in outbred populations, a combination of identity‐by‐descent and identity‐by‐state is the norm. However, with increased levels of endogamy in the population, recurrent sampling of the identical‐by‐descent deletion alleles is more likely.

We also identify the reciprocal duplication breakpoint generated by NAHR within this region from analysis of the fosmid sequence, as predicted by the NAHR model. We found three gene conversion alleles within the region that contains the *FCGR3B* deletion breakpoints. It is likely that the longer region of gene conversion is more likely to have been generated by a double crossover event, although long gene‐conversion tracts are known to exist [Chen et al., 2007; Hallast et al., 2013].

The *FCGR3B* deletion allele has been shown to be associated with RA in several studies. In some studies, the association is with all forms of RA [McKinney et al., 2010; Graf et al., 2012]; in others, the effect is more pronounced in autoantibody‐positive RA [Robinson et al., 2012]. However, other studies failed to find an association [Chen et al., 2007; Mamtani et al., 2010; Marques et al., 2010], and all studies have been limited by noisy typing of *FCGR3B* copy number and/or small sample sizes that are likely to be underpowered. In this study, we replicate an association of the *FCGR3B* deletion allele with RA (Fisher's exact test, *P* = 2.9×10^−3^) in a large cohort of 1,982 cases and 3,271 controls, which is three times the size of the largest previous study. Furthermore, an odds ratio of 1.61 (95% CI 1.16–2.24) is a size of effect consistent with previous studies.

Nevertheless, this study has limitations. First, the distinction between *FCGR3A* and *FCGR3B* deletions using aCGH data is not absolutely clear, so there may be some misclassification of *FCGR3A* and *FCGR3B* deletions where signals are at the boundary of the two Gaussian curves in the mixture model. Second, as we have shown, *FCGR3B* deletion alleles can have different breakpoints in the UK population, and it is probable that structurally different alleles will have different effects on RA risk. Indeed, a previous study has suggested that the association of inflammatory disease with *FCGR3B* CN may not be due to the loss of *FCGR3B* as such, but rather the juxtaposition of *FCGR2C* regulatory elements next to a full *FCGR2B* coding sequence, causing ectopic expression of the inhibitory receptor *FCGR2B* on NK cells [Mueller et al., 2012]. If this is correct, one or more PSVs distinguishing A from B paralog repeats must lead to ectopic expression on NK cells, but these functional sequence changes have not yet been identified. An alternative explanation for the association is that certain deletion breakpoints generate *FCGR2C/B* fusion genes, including a *FCGR2B* null variant [Nagelkerke et al., 2015]. We have shown that upstream and coding regions of both *FCGR2B* and *FCGR2C* are affected by gene‐conversion events, and the role of gene conversion in modulating variation in gene expression in deleted and nondeleted chromosomes remains to be determined.

Recent studies have shown that some structural variation can be imputed from flanking dense SNP haplotypes [Sekar et al., 2016]. This enables reanalysis of huge cohorts that have undergone genome‐wide SNP typing, indirectly imputing structural variants and testing those imputed variants for association with a phenotype. While there is no simple tagging SNP for *FCGR3B* deletion alleles [Hollox et al., 2009], it is possible that copy‐number and gene‐conversion alleles can be indirectly imputed from flanking SNP haplotypes. If so, the nature of the association between genetic variation at this locus and RA, as well as other autoimmune diseases, can be resolved.

## Supporting information

Supplementary Figure 1 Deletion breakpoints, gene conversion regions and other features involving *FCGR2B/FCGR2C*
Supplementary table 1 8‐SNP Haplotype composition
Supplementary table 2 Frequency of 8‐SNP haplotypes in Human Genome Diversity project populations
Supplementary table 3. Haplotypes of individuals homozygous for *FCGR3B* deletion
Supplementary table 4 Association analysis of *FCGR3A* deletion and RA
