# Supplementary material for: Understanding the Genomic Structure of Copy‐Number Variation of the Low‐Affinity Fcγ Receptor Region Allows Confirmation of the Association of FCGR3B Deletion with Rheumatoid Arthritis
Source: Hum Mutat. 2017 Feb 15;38(4):390–9. doi: 10.1002/humu.23159 (PMC5363352; doi:10.1002/humu.23159)
Supplement: Supplementary file 1 — Supplementary Figure 1 Deletion breakpoints, gene conversion regions and other features involving FCGR2B/FCGR2C Supplementary table 1 8‐SNP Haplotype composition Supplementary table 2 Frequency of 8‐SNP haplotypes in Human Genome Diversity project populations Supplementary table 3. Haplotypes of individuals homozygous for FCGR3B deletion Supplementary table 4 Association analysis of FCGR3A deletion and RA [file HUMU-38-390-s001.docx]

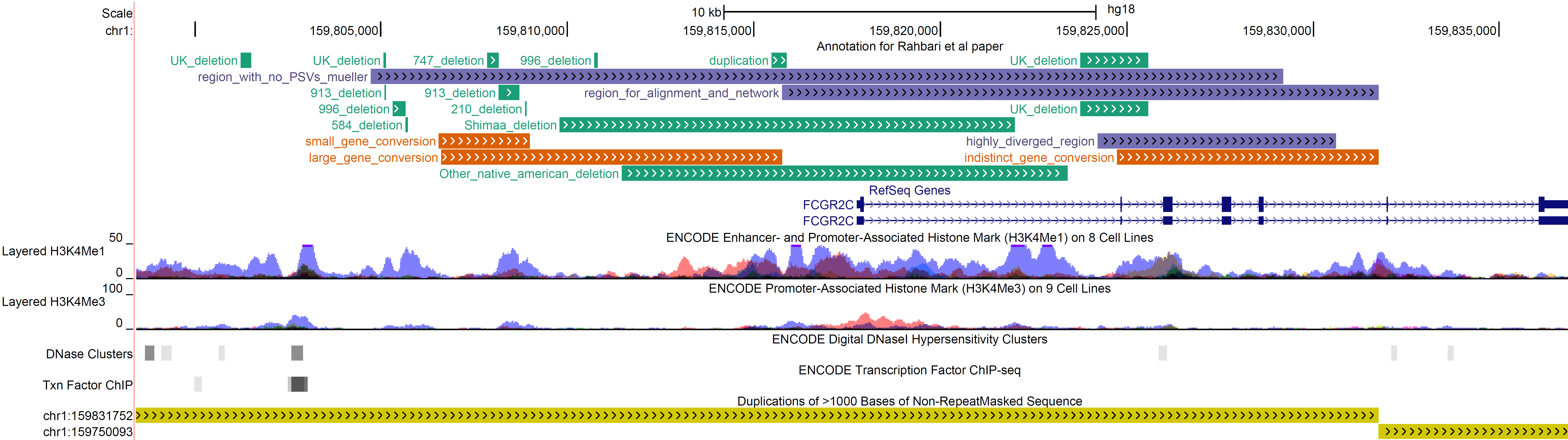


**Supplementary Figure 1 Deletion breakpoints, gene conversion regions and other features involving *FCGR2B/FCGR2C***

The UCSC Genome Browser for human genome assembly hg18 (http://genome.ucsc.edu), annotated with key regions discussed in this paper. The selected region corresponds to the proximal repeat, and the boundary with the distal repeat, shown as two different duplications in the lowermost track. The annotations, although shown as annotated on the proximal repeat, refer to both proximal and distal repeats – for example the green annotations indicating deletion breakpoints show the region where an NAHR event occurred between the proximal and distal repeats that generated a deletion allele. The orange annotations refer to regions of gene conversion, and purple annotations refer to other regions discussed in the paper. The *FCGR2C* gene is annotated, as it bridges the proximal and distal repeats at this point. Indicators of regulatory regions, as identified by the ENCODE project, are also annotated – for full details refer to <http://genome.ucsc.edu>.

**Supplementary table 1 8-SNP Haplotype composition**

| **Haplotype** | **rs1050501** | **rs723177** | **rs1256286** | **rs11801845** | **rs1832739** | **rs6697139** | **rs1340976** | **rs4657090** |
| --- | --- | --- | --- | --- | --- | --- | --- | --- |
| **Ancestral** | T | C | C | G | G | G | C | G |
| **A** | C | C | C | G | G | G | A | G |
| **K** | T | C | C | G | G | G | A | G |
| **M** | T | C | C | T | G | G | A | G |
| **R** | T | C | T | G | T | G | C | G |
| **T** | T | C | T | G | T | T | A | G |
| **V** | T | T | C | G | G | G | A | A |

**Supplementary table 2 Frequency of 8-SNP haplotypes in Human Genome Diversity project populations (see Machado et al. 2012)**

| **Haplotype** | **East Asia** | **Europe** | **Middle East** | **South Asia** | **Sub Saharan Africa** | **South America** |
| --- | --- | --- | --- | --- | --- | --- |
| A | 0.21 | 0.14 | 0.13 | 0.14 | 0.26 | 0.00 |
| K | 0.01 | 0.10 | 0.13 | 0.08 | 0.16 | 0.01 |
| M | 0.02 | 0.04 | 0.06 | 0.08 | 0.16 | 0.01 |
| R | 0.49 | 0.22 | 0.13 | 0.24 | 0.07 | 0.76 |
| T | 0.01 | 0.22 | 0.24 | 0.18 | 0.02 | 0.13 |
| V | 0.24 | 0.25 | 0.25 | 0.22 | 0.20 | 0.07 |
| others | 0.02 | 0.03 | 0.06 | 0.06 | 0.12 | 0.02 |

Note: totals do not always add to 1 because of rounding error.

**Supplementary table 3. Haplotypes of individuals homozygous for *FCGR3B* deletion**

| **Sample** | **Population** | **FCGR3A** | **FCGR3B** | **SNP Haplotypes** | **Reference** |
| --- | --- | --- | --- | --- | --- |
| HGDP00210 | Sindhi | 2 | 0 | KT | Machado *et al* 2012 |
| HGDP00913 | Mandenka | 2 | 0 | AM | Machado *et al* 2012 |
| HGDP00996 | Karitiana | 2 | 0 | RR | Machado *et al* 2012 |
| CO092 | British | 2 | 0 | KV | This paper |
| UKTS8802 | British | 2 | 0 | KR | This paper |
| ASH551 | Ashaninka | 2 | 0 | RR | This paper |
| ASH882 | Ashaninka | 2 | 0 | RR | This paper |
| SH015 | Matsiguenga | 2 | 0 | RR | This paper |
| SH221 | Matsiguenga | 2 | 0 | RR | This paper |
| SH236 | Matsiguenga | 2 | 0 | RR | This paper |
| SH497 | Matsiguenga | 2 | 0 | RR | This paper |
| ASH534 | Ashaninka | 2 | 0 | RR | This paper |
| SH01 | Matsiguenga | 2 | 0 | RR | This paper |

**Supplementary table 4 Association analysis of *FCGR3A* deletion and rheumatoid arthritis**

| **Genotype** | **RA** | **Control** | **OR (95% confidence intervals)** | **P value** |
| --- | --- | --- | --- | --- |
| ***FCGR3A* deletion carriers** | 84 | 130 | 1.02(0.76-1.36) | 0.886 |
| **Non-carriers** | 1898 | 3141 |  |  |
